# Temporal and spatial dynamic propagation of electroencephalogram by combining power spectral and synchronization in childhood absence epilepsy

**DOI:** 10.3389/fninf.2022.962466

**Published:** 2022-08-16

**Authors:** Lisha Zhong, Jiangzhong Wan, Jia Wu, Suling He, Xuefei Zhong, Zhiwei Huang, Zhangyong Li

**Affiliations:** ^1^School of Communication and Information Engineering, Chongqing University of Posts and Telecommunications, Chongqing, China; ^2^School of Medical Information and Engineering, Southwest Medical University, Luzhou, China; ^3^Children’s Hospital of Chongqing Medical University, Chongqing, China; ^4^Central Nervous System Drug Key Laboratory of Sichuan Province, Luzhou, China; ^5^Research Center of Biomedical Engineering, Chongqing University of Posts and Telecommunications, Chongqing, China

**Keywords:** childhood absence epilepsy, EEG, power spectral density, phase locking value, time-frequency-spatial characteristics

## Abstract

**Objective:**

During the transition from normal to seizure and then to termination, electroencephalography (EEG) signals have complex changes in time-frequency-spatial characteristics. The quantitative analysis of EEG characteristics and the exploration of their dynamic propagation in this paper would help to provide new biomarkers for distinguishing between pre-ictal and inter-ictal states and to better understand the seizure mechanisms.

**Methods:**

Thirty-three children with absence epilepsy were investigated with EEG signals. Power spectral and synchronization were combined to provide the time-frequency-spatial characteristics of EEG and analyze the spatial distribution and propagation of EEG in the brain with topographic maps. To understand the mechanism of spatial-temporal evolution, we compared inter-ictal, pre-ictal, and ictal states in EEG power spectral and synchronization network and its rhythms in each frequency band.

**Results:**

Power, frequency, and spatial synchronization are all enhanced during the absence seizures to jointly dominate the epilepsy process. We confirmed that a rapid diffusion at the onset accompanied by the frontal region predominance exists. The EEG power rapidly bursts in 2–4 Hz through the whole brain within a few seconds after the onset. This spatiotemporal evolution is associated with spatial diffusion and brain regions interaction, with a similar pattern, increasing first and then decreasing, in both the diffusion of the EEG power and the connectivity of the brain network during the childhood absence epilepsy (CAE) seizures. Compared with the inter-ictal group, we observed increases in power of delta and theta rhythms in the pre-ictal group (*P* < 0.05). Meanwhile, the synchronization of delta rhythm decreased while that of alpha rhythm enhanced.

**Conclusion:**

The initiation and propagation of CAE seizures are related to the abnormal discharge diffusion and the synchronization network. During the seizures, brain activity is completely changed with the main component delta rhythm. Furthermore, this article demonstrated for the first time that alpha inhibition, which is consistent with the brain’s feedback regulation mechanism, is caused by the enhancement of the network connection. Temporal and spatial evolution of EEG is of great significance for the transmission mechanism, clinical diagnosis and automatic detection of absence epilepsy seizures.

## Introduction

Childhood absence epilepsy (CAE) is the most common childhood epilepsy syndrome in school-aged children, accounting for 10–17% of all pediatric epilepsy (Matricardi et al., 2014). CAE is clinically characterized by frequent and transient impairment of consciousness (with sudden onset and offset) and is accompanied by staring, spontaneous eye-opening and random eye-blinking (Hirsch and Marescaux, 1994; Panayiotopoulos, 1997). The duration of the typical CAE is about 5–30 s, with several to hundreds of attacks per day (Penry et al., 1975). CAE, as age-dependent epilepsy, has long been labeled “benign” because children usually achieve an optimistic recovery with appropriate treatment (Tashkandi et al., 2019). However, comorbidities such as cognitive, behavioral, emotional disorders, and language barriers have been reported in previous research (Caplan et al., 2008; Vega et al., 2011; Verrotti et al., 2015). Due to the high frequency and comorbidities of CAE, it can seriously affect children’s lives. Electroencephalography (EEG) is an essential tool for clinical diagnosis and treatment of epilepsy with advantages of non-invasive, high time resolution and sustainable monitoring, which is already widely used to detect seizures (Gao and Hu, 2013; Zhong et al., 2022). Ictal EEG in CAE is characterized by a high-amplitude, generalized, bilateral, symmetrical, and synchronous discharges of 3 Hz spike-and-waves, while the inter-ictal EEG typically shows a normal background activity (Fisher et al., 2017). The pathophysiology of CAE has been extensively carried out in both animal and human models over the past decade (Meeren et al., 2005; Sitnikova and Luijtelaar, 2006; van Luijtelaar et al., 2011). Dynamic changes in the spatiotemporal course have been studied in CAE using EEG (Kokkinos et al., 2013, 2017; Leitgeb et al., 2020). However, most studies have focused on the generation and propagation of ictal spike-wave discharges during seizures. Especially, quantitative biomarkers from the temporal and spatial evolution of EEG remains elusive. Therefore, this article not only analyzes the time-frequency-spatial characteristics of EEG during the whole process from normal to seizure states, but also further investigates some quantitative EEG markers to distinguish among normal, pre-ictal, and ictal states, which is helpful for the diagnosis and early warning of CAE seizures.

Power Spectrum has been applied to explore the intracranial EEG patterns in 15 patients with partial epilepsy as early as 1995 (Alarcon et al., 1995). Spectral power of interictal EEG was utilized as a biomarker for the diagnosis and treatment of idiopathic generalized epilepsy (Pegg et al., 2020). A device was designed to predict seizures using relative power spectral of EEG and provide responsive electrical stimulation feedback to interrupt seizures (Bandarabadi et al., 2015). Some other researchers (Park et al., 2011; Zhang and Parhi, 2016; Peng et al., 2021) extracted power spectral and the ratio of power spectral and combined it with machine learning to predict seizures. In previous studies (Park et al., 2011; Zhang and Parhi, 2016; Pegg et al., 2020; Peng et al., 2021), power spectral density (PSD) has been proven effective in detecting or predicting seizures. In this article, PSD is conducted to explore the temporal and spatial evolution of CAE EEG signals combined with the topographic map.

In terms of spatial evolution, CAE is caused by synchronous, generalized discharges, making it of great importance to pay more attention to synchronization. Previous researches (van Luijtelaar and Sitnikova, 2006; Westmijse et al., 2009; Gupta et al., 2011; Gadad et al., 2018) have demonstrated that CAE seizures originate from an activated brain area at the onset, spread to other brain regions, and interact with each other, resulting in abnormal synchronous discharges throughout the brain network. Synchronization which was used to quantify interactions between different areas of the brain has been reported in van Diessen et al. (2013), van Mierlo et al. (2014), and Yaffe et al. (2015). The brain is considered to be a nonlinear dynamical system, and the phase locking, as a nonlinear method, can be used to analyze the nonlinear, non-stationary and chaotic systems (Aydore et al., 2013). In addition, phase locking value (PLV) is amplitude-independent (Piqueira, 2011), making it more suitable for analyzing the EEG signals that are affected by synchronous amplitude changes caused by eye movements and other activities (Wang et al., 2020). Therefore, this article employs PLV to assess the synchronization between a pair of electrodes as the spatial characteristic of EEG and construct the dynamic brain network connectivity.

In summary, PSDs and PLVs of the EEG and its rhythms including the delta (1–4 Hz), theta (4–8 Hz), alpha (8–12 Hz), beta (12–30 Hz), and gamma (30–70 Hz) bands are calculated in this article. Combined with the topographic map, this article realizes to visualize the spatial distribution of PSD and dynamic network connectivity over time. To better understand the dynamic changes and evolutionary patterns, a comparative study of inter-ictal, pre-ictal, and ictal states was carried out, which also helps to explore quantitative biomarkers that distinguish among these three states. Therefore, our research is beneficial for providing important EEG markers for the detection and early warning of CAE seizures in the clinical applications.

## Materials and methods

### Patients

In this study, we included 33 children (23 female and 10 male) with absence epilepsy. The age of the children ranged between 5 and 12 years old (8.34 ± 2.17 years). The inclusion criteria were as bellow: (1) diagnosed as absence epilepsy according to the International League Against Epilepsy (ILAE); (2) the duration of seizures lasted ≥4 s in EEG recordings with 3–4 Hz bilateral and synchronous spike and wave discharges; (3) anti-seizure medication (ASM) has not been used in these CAE patients; and (4) no other seizure types present and normal neurologic development. The demographic data of these recruited patients were collected from the Children’s Hospital of Chongqing Medical University between December 2015 and October 2017. This study was conducted by the Declaration of Helsinki for experimentation in humans and was performed with informed consent obtained from all subjects’ parents after approval of the Ethical Committee of the Children’s Hospital of Chongqing Medical University. The detailed clinic information of the CAE patients are shown in Table 1.

**TABLE 1:** Detailed information of the clinical CAE patients.

Patients	Age (years)	Gender	No. of seizures	Seizures are induced by hyperventilation or spontaneous	Brain region of seizure origin
1	11.42	Male	1	Spontaneous	Right frontal region
2	6.42	Female	4	Hyperventilation	Frontal lobes
3	11.67	Male	1	Hyperventilation	Prefrontal lobe
4	5.92	Female	5	Spontaneous	Frontal lobe
5	6.25	Female	2	Hyperventilation	Prefrontal lobe
6	5.92	Female	2	Spontaneous	Frontal lobe
7	6.67	Male	1	Hyperventilation	Whole brain (mainly the left central, occipital, and middle temporal regions)
8	6.42	Female	6	Hyperventilation	Occipital region
9	9	Male	3	Hyperventilation	Frontal lobe
10	8.08	Female	1	Hyperventilation	Prefrontal region
11	8.92	Male	1	Hyperventilation	Left prefrontal lobe
12	9.33	Female	1	Hyperventilation	Right mid-posterior temporal region
13	11.33	Male	1	Spontaneous	Not found
14	8.92	Female	3	Hyperventilation	Prefrontal lobe
15	8.5	Female	1	Hyperventilation	Frontal lobe
16	6.17	Female	3	Spontaneous	Frontal and temporal lobes
17	5.25	Female	1	Hyperventilation	Not found
18	6	Female	3	Hyperventilation	Right middle temporal region
19	9	Female	1	Spontaneous	Frontal lobe
20	7.58	Male	1	Hyperventilation	Prefrontal, frontal, and lateral frontal regions
21	8.75	Female	1	Hyperventilation	Not found
22	10.75	Male	2	Spontaneous	Frontal and temporal lobes
23	12.75	Female	5	Hyperventilation	Frontal lobe
24	6	Female	3	Spontaneous	Frontal lobe
25	10	Female	2	Hyperventilation	Prefrontal region
26	10.25	Female	2	Spontaneous	Frontal and temporal lobes
27	9.42	Male	1	Hyperventilation	Prefrontal, frontal, and parafrontal regions
28	10	Female	1	Hyperventilation	Not found
29	6.33	Female	1	Hyperventilation	Left parietal region
30	6.83	Male	1	Spontaneous	Left occipital and posterior temporal regions
31	12.5	Female	1	Hyperventilation	Not found
32	6.58	Female	4	Hyperventilation	Frontal lobe
33	6.42	Female	1	Hyperventilation	Prefrontal and parafrontal regions
Total	8.34 ± 2.17	23 females, 10 males	67	21 seizures are occurred spontaneously and 46 seizures are induced by hyperventilation	22 patients with seizures originating from frontal-related regions (including frontal, prefrontal, lateral frontal, right frontal, left prefrontal lobes, etc.)

### Data recordings

**FIGURE 1:**
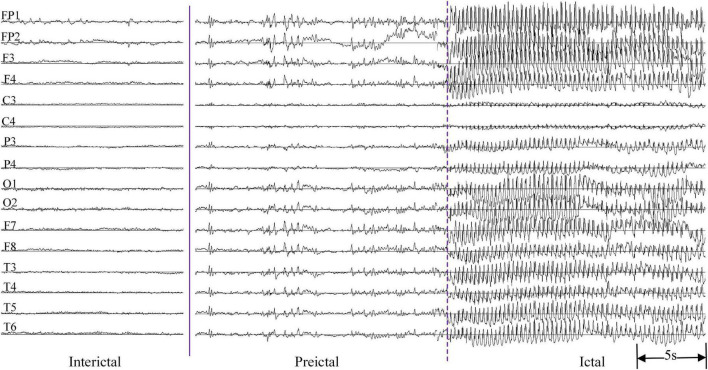
An example of multi-channel EEG signals in inter-ictal, pre-ictal, and ictal states.

The EEG was recorded by the Neurofax EEG-1200 version (Nihon Kohden, Japan) system with a sampling frequency of 200 Hz. In total, 16 Ag/AgCl electrodes were placed on the scalp (Fp1, Fp2, F7, F8, F3, F4, C3, C4, P3, P4, T3, T4, T5, T6, O1, and O2) according to the international 10–20 system, using monopolar montage with average reference. The impedances of all EEG electrodes were kept below 10 kΩ. At least one clinical absence epilepsy seizure should be recorded. If there was no spontaneous seizure during the process of recording the EEG signals, hyperventilation was performed to provoke a seizure. Inter-ictal EEG acquisition lasted about 5 min, and the duration of EEG was approximately 20 min including hyperventilation. Multiple consecutive seizures occurred in some EEG recordings. A total of 67 absence seizure events were recorded in 33 patients with a minimum of one seizure and a maximum of six seizures. All the seizures were annotated by physicians with the duration ranging from 9 to 32 s. The EEG signals without artifacts in the 30 s before epileptic seizures were selected as the pre-ictal group. In the inter-ictal state, 30 s background EEG data were chosen as the normal control group. Figure 1 is an example of multi-channel EEG signals in inter-ictal, pre-ictal and ictal states, respectively.

### Methods of electroencephalography signals analysis

**FIGURE 2:**
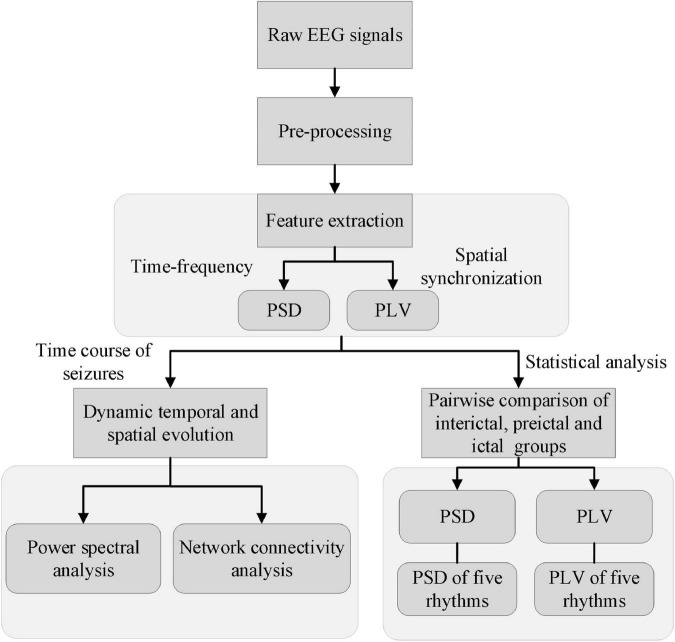
A schematic illustration shows the detailed steps of the study.

Figure 2 shows a schematic illustration of the detailed steps. First, the raw EEG signals were filtered by using a fourth-order Butterworth bandpass filter (1–70 Hz) and a notch filter (50 Hz) to reduce noise. Artifacts such as eye movement, eye blink, muscle artifact were removed by using independent component analysis (ICA) in the EEGLAB toolbox (Delorme and Makeig, 2004) with the guidelines (Urigüen and Garcia-Zapirain, 2015). Note that artifacts that could not be removed by signal processing, such as severe crying or head movements, were directly excluded from the experimental sample data. The clean EEG signals were divided into 1-s epochs with a group of 1 s non-overlapping moving windows. The reason is that literatures (Gupta et al., 2011; Wu et al., 2017) have reported that the duration of 1-s is always corresponding to the spike discharges and is essential for the network connectivity synchronization. Then calculate the PSD and PLV of these artifact-free epochs. Third, the time course of these features (i.e., PSD and PLV) was analyzed to explore the dynamic temporal and spatial evolution of EEG signals during the whole process of the seizures. Lastly, PSDs and PLVs of EEG and each rhythm were compared pairwise between the three groups: inter-ictal group, pre-ictal group, and ictal group. EEG analysis was performed with the software package MATLAB R2016b (The MathWorks, Inc., Natick, MA, United States) and its EEGLAB and statistics toolbox.

#### Power spectral analyses

As a non-parametric method based on periodogram, the Welch method is superior to other parametric approaches due to the availability of efficient Fast Fourier transform algorithms (Zhang and Parhi, 2016), with the advantages of fast calculation speed and multiple windows for selection. In accordance with Welch’s periodogram method, the PSD of EEG signals in each frequency band were calculated by using 1-s hamming windows. The PSD can be estimated by the following steps (Welch, 1967):

First, the signal _x(n),n={0,1…N–1}_ is divided into L segments. Each segment has M points and the data in the *i*-th segment can be expressed:


(1) $$x_{\text{i}}\,\left(n\right)=x\,\left(n+i\,M-M\right),0\leq n\leq \text{M},1\leq i\leq L$$

Then take the fast Fourier transforms of these sequences and the *i*-th periodogram is:


(2) $$I_{\text{i}}\,\left(\omega \right)=\frac{1}{U}\,{\left|\sum_{n=0}^{M-1}x_{i}\,\left(n\right)\,w\,\left(n\right)\,e^{-j\,w\,n}\right|}^{2},i=1,2\,\ldots ,M-1$$

where $U=\frac{1}{M}\,\sum_{n=0}^{M-1}w^{2}\,\left(n\right)$.

Finally, the PSD is obtained as:


(3) $$P\,S\,D\,\left(e^{\text{jw}}\right)=\frac{1}{L}\,\sum_{i=1}^{L}I_{\text{i}}\,\left(\omega \right)$$

The average power spectrum can be calculated as:


(4) $$E\,\left[P\,S\,D\,\left(e^{\text{jω}}\right)\right]=\frac{1}{2\,\pi }\,\int_{-\pi }^{\pi }P_{\text{xx}}\,\left(e^{\text{jw}}\right)\,W\,\left(e^{j\,\left(n-w\right)}\right)\,d\,w$$

where


(5) $$W\,\left(e^{\text{jω}}\right)=\frac{1}{M\,U}\,{\left|\sum_{n=0}^{M-1}w\,\left(n\right)\,e^{j\,\omega }\right|}^{2}$$

(6) $$P_{\text{xx}}\,\left(e^{j\,\omega }\right)=\sum_{m=-\infty }^{\infty }r_{x}\,\left(m\right)\,e^{-j\,\omega \,m}$$

*r*_*x*_(*m*) is the autocorrelation function of the *x*(*n*).

Mathematically, the spectral power in the frequency bands (delta, theta, alpha, beta, and gamma) is calculated as (Zhang and Parhi, 2016):


(7) $$P_{\text{i}}=\log\sum_{\omega \in b\,a\,n\,d\,i}PSD_{\text{s}}\left(\omega \right),\left\{i=delta,theta,alpha,beta,gamma.\right\}$$

#### Synchronization analyses

Phase locking value, as an independent of amplitude, is suitable to measure the phase synchronization of EEG signals, which can be computed as follow (Lachaux et al., 2015):


(8) $$P\,L\,V\,\left(t,f\right)=\frac{1}{N}\,\left|\sum_{n=1}^{N}\text{exp}\,\left(j\,\left\{\Delta \,\Phi \,\left(t,f\right)\right\}\right)\right|$$

where ΔΦ(*t*,*f*) is the instantaneous phase difference between a pair of EEG channels at time t and frequency *f*. Taking channels 1 and 2 for example, ΔΦ(*t*,*f*) is calculated as:


$$\Delta \,\Phi \,\left(t,f\right)=\Phi_{\text{ch}\,1}\,\left(t,f\right)-\Phi_{\text{ch}\,2}\,\left(t,f\right)\,\left(8\right)$$

where Φ_ch1_(*t*,*f*) and Φ_ch2_(*t*,*f*) are the instantaneous phases of the EEG signals in channel 1 and channel 2, respectively. Instantaneous phase ϕ(*t*) is obtained by the Hilbert transform. The absolute value for PLVs ranges from 0 to 1, where 0 corresponds to no synchrony and 1 corresponds to maximum synchrony. In this article, PLVs were converted into binary matrices to construct the dynamic brain network by setting PLV >0.6, which means a strong correlation.

### Statistical analysis

After assessment of normality, a Kruskal–Wallis H-test was used to compare the EEG PSD and PLV of five frequency bands (delta, theta, alpha, beta, and gamma) across the three groups (inter-ictal, pre-ictal, and inter-ictal groups). *P* < 0.05 indicates a statistically significant difference (two-sided). All statistical analyses and computations were performed in SPSS version 22.0 for Windows software (SPSS Inc., Chicago, IL, United States).

## Results

Results include two parts: (1) the time course of the variables (PSD and PLV) during the whole process of a seizure to explore the dynamic temporal and spatial evolution of EEG signals and the common characteristics that exist during the CAE seizures for all patients. (2) In order to better discover EEG biomarkers and understand the dynamic mechanism of epilepsy, the statistical analysis to compare among inter-ictal, pre-ictal, and ictal groups in EEG power spectral and synchronization.

### Dynamic temporal and spatial evolution

#### Power spectral analysis

**FIGURE 3:**
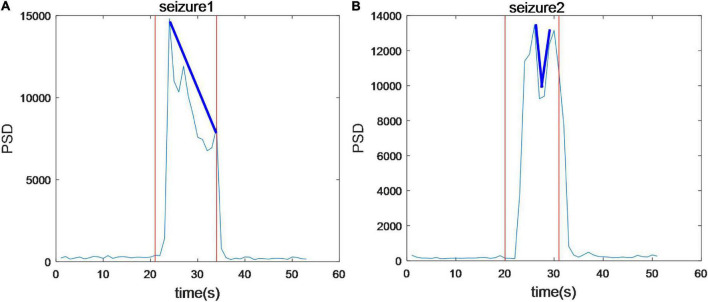
The average power spectral density of 16 channels during seizures. The trend of PSD in two seizures in the same patient is different.

Time-frequency analysis of epileptic EEG signals was carried out by a series of 1s time-scale power spectral constructed by PSD. We found that for all seizures power in 2–4 Hz is the highest especially in 3 Hz. Both the high frequency and EEG power increase extremely during the seizures, but delta as the slow-wave rhythm is the main component of EEG signals. To further analyze the trend of PSD during seizures, the average PSD of 16 channels was calculated, as shown in Figure 3. Figure 3A shows that average PSD increases rapidly at the onset, reaching the maximum within 3 s, but then gradually decreases during this seizure (shown in blue line). However, the average PSD of the subsequent seizure in this sample decreases and then increases as shown in Figure 3B. These results show that the trend of PSD in two seizures for the same patient is different. In general, no gradual increase occurs throughout the seizure process in this experiment.

**FIGURE 4:**
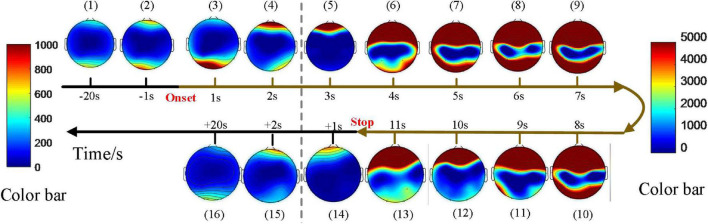
An example of the topographic distribution of the EEG power. Color bar scale of the six topographies on the left of the gray dotted line is 0–1,000 and the scale of the rest topographies on the right is 0–5000 due to an enormous increase in PSD during seizures. –20 s: The average of PSD in 20 s before seizure start; +20 s: The average of PSD in 20 s after seizure stopped. There was a process of first increasing and then decreasing in the spatial distribution of PSD for all seizures.

Dynamic spatial propagation of EEG power was combined with topography, as shown in Figure 4. The distribution of the PSD before the seizure onset is almost the same as that after the seizures (subfigure 1 vs. subfigure 16), and PSD of the whole brain region is low at these moments. PSD in the seizure state is much higher and distributed more widely than that in the pre-ictal and post-ictal states. The pattern of spatial evolution in this sample was first transmitted to the prefrontal lobe (subfigure 4 and subfigure 5), spread to the upper part of the brain (subfigure 6), then covered the most range of the brain (subfigures 7–10), finally gradually decreased until the seizures stopped (subfigures 11–13). Note that the topographic map can not represent the power over time. The average PSD in this sample tends to decrease gradually during this seizure (Figure 3A). Still, there is a process of first increasing and then decreasing in the spatial distribution, indicating that the evolution of power and distribution is inconsistent. For a total of three seizures in this patient, the average PSDs varies in trend, while the spatial distribution evolution is consistent.

#### Network connectivity synchronization analysis

**FIGURE 5:**
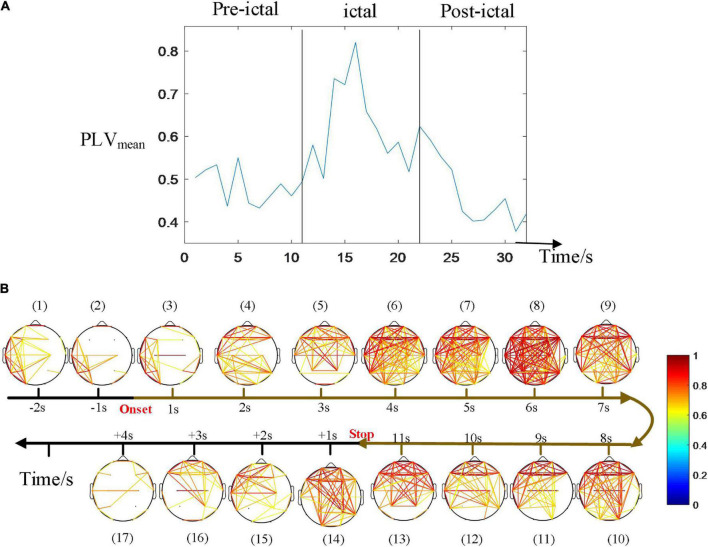
Time courses of the synchronization. **(A)** The average of PLV during the seizure. **(B)** Dynamic network connectivity distributions. The strongest connectivity in **(B)** just corresponded to the maximum value of the PLV_mean_ (at 6 s after onset in this seizure). The pattern of the evolution of network synchronous connectivity is consistent with that of the spatial diffusion range, both first increasing and then decreasing.

Figure 5 depicts an example of the time courses of synchronization and dynamic network connectivity. With the development of this CAE seizure, synchronization and network connectivity show a progressive increase at the onset, then extend to a wider range of brain regions, reaching its maximum at 6 s, and finally decrease. We found that the strongest connectivity (subfigure 8 of Figure 4B) just corresponds to the maximum value of the PLV_mean_. At this moment, comprehensive connections have been observed, indicating a generalized seizure throughout the brain. Moreover, the pattern of the evolution of network synchronous connectivity is consistent with the spatial diffusion range (Figure 4), both first increasing and then decreasing.

### Comparison among inter-ictal, pre-ictal, and ictal groups in electroencephalography power spectral and synchronization

#### Spatial distribution and rhythms analysis of electroencephalography power spectral

**FIGURE 6:**
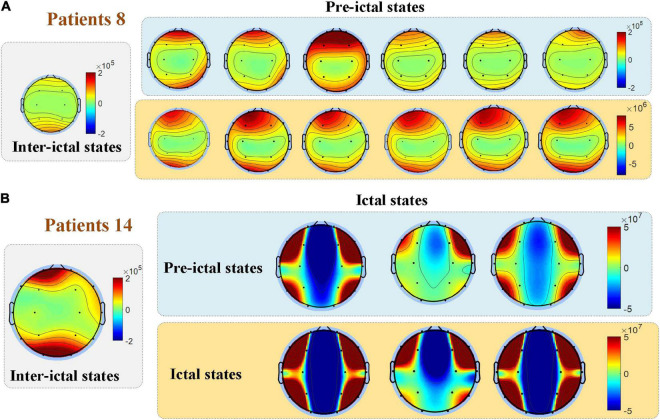
Comparisons among the three groups in the spatial distribution of EEG power. The spatial distribution of EEG PSD varies among individuals, while most patients have a similar spatial distribution of multiple consecutive seizures as shown in **(A)**. **(B)** For some patients, the spatial distribution of the pre-ictal state showed similarities to the ictal state.

Figure 6 compares the spatial distribution through single-subject’s PSD topographies in inter-ictal, pre-ictal, and ictal groups. PSD topographies of all samples are provided in Supplementary material. The experimental results show that EEG PSD’s spatial distribution varies greatly among individuals. Compared to the pre-ictal and inter-ictal groups, PSD in the ictal group was significantly enhanced in the entire brain. We also found that most patients have the similar spatial distribution of multiple consecutive seizures. Compared to the inter-ictal state, EEG power spectral in the pre-ictal group is higher than that in the inter-ictal group, and the spatial distribution has also changed. More importantly, compared to the ictal period, the PSD value was much lower in the preictal period; nevertheless, the spatial distribution showed a similarity for some patients.

**FIGURE 7:**
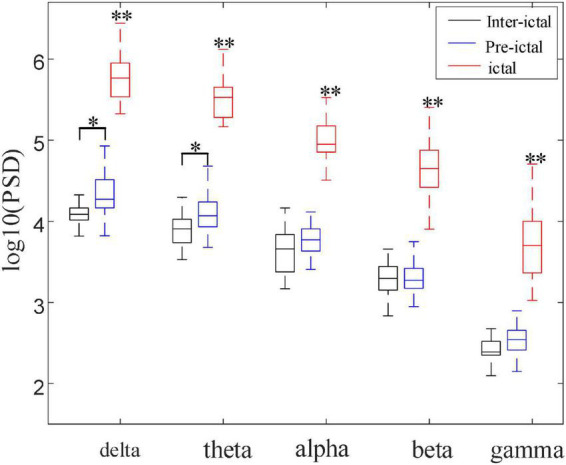
Comparisons among the three groups of PSD in five frequency bands (*P < 0.05, **P < 0.001). These five frequency bands (delta, theta, alpha, beta, and gamma) in the ictal group were significantly increased compared with pre-ictal and inter-ictal groups, respectively (*H*_*delta*_ = 103.259, *H*_*theta*_ = 103.842, *H*_*alpha*_ = 100.364, *H*_*beta*_ = 101.648, *H*_*gamma*_ = 100.785, *P* < 0.001). Compared with inter-ictal group, delta and theta rhythms were significantly increased in the pre-ictal group (*H*_*delta*_ = 17.132, *P* = 0.042; *H*_*theta*_ = 18.403, *P* = 0.036) with the degrees of freedom *m* = 2.

Figure 7 compares the different rhythmic activities (delta, theta, alpha, beta, and gamma) among the inter-ictal, pre-ictal, and ictal groups. As the frequency gradually increases from delta, theta, alpha, beta to gamma rhythm, PSD shows a decreasing trend in these three groups. The statistical results show that all the frequency bands in the ictal group are significantly increased compared to the other two groups (*P* < 0.001, *P* < 0.001). Although high-frequency components do enormously increase in the ictal group, low-frequency components, delta band, are still dominant. In contrast to the inter-ictal group, the delta and theta rhythms in low-frequency bands are significantly increased in the pre-ictal group (*H* = 103.259, *P* = 0.042; *H* = 103.842, *P* = 0.036) with the degrees of freedom *m* = 2, but there is no significant difference in alpha, beta, and gamma rhythms.

#### Comparisons among the three groups in synchronization

**FIGURE 8:**
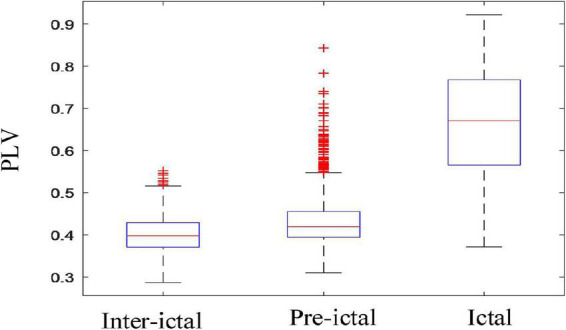
Comparisons among the three groups in synchronization (inter-ictal vs. pre-ictal, inter-ictal vs. ictal, and pre-ictal vs. ictal). Boxplots of PLV in three groups; PLV was extremely heightened during the seizures. There were much more outliers in the pre-ictal group than inter-ictal group (the red “+” represents outliers).

Figure 8 shows the comparison among inter-ictal, pre-ictal, and ictal groups of PLV in synchronization. PLV is extremely heightened during seizures, and the main range of its values (the interquartile of the boxplot) only overlapped with the outliers in the pre-ictal group, are much larger than that in the other two groups. The boxplots between the pre-ictal and the inter-ictal groups are similar, with a slightly higher median in the pre-ictal group. The obvious difference is that there were much more outliers in the pre-ictal group (the red “+” represents outliers).

**FIGURE 9:**
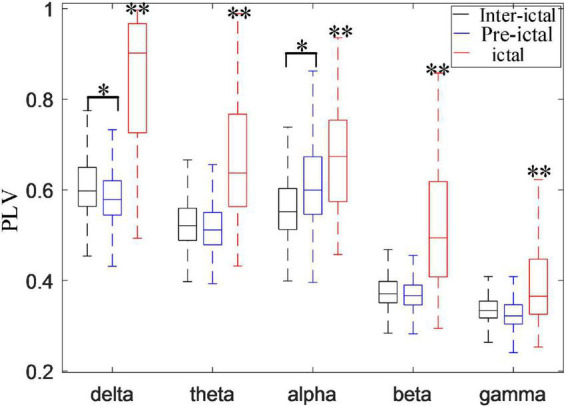
Comparisons among inter-ictal, pre-ictal, and ictal groups in synchronization of five frequency bands (*P < 0.01, **P < 0.001). The synchronization of all the rhythms in the ictal group was higher than inter-ictal and pre-ictal groups (*P* < 0.001). Compared with the inter-ictal group, the synchronization of the delta band in the pre-ictal group was significantly decreased (*H*_*delta*_ = 18.569, *P* < 0.05), while the alpha band is enhanced (*H*_*alpha*_ = 29.517, *P* < 0.01) with the degrees of freedom *m* = 2.

Figure 9 compares the synchronization of these five rhythms among the inter-ictal, pre-ictal and ictal groups. The synchronization of all the rhythms in the ictal group is obviously higher than that in the other two groups (*P* < 0.001, *P* < 0.001). Compared to the inter-ictal group, the synchronization of the delta band in the pre-ictal group is significantly decreased (*H*_*delta*_ = 18.569, *P* < 0.05), while the alpha band is enhanced (*H*_*alpha*_ = 29.517, *P* < 0.01). There is no significant difference in theta, beta, and gamma rhythms between pre-ictal and inter-ictal groups.

## Discussion

This study aimed to provide biomarkers through analysis time-frequency-spatial information of EEG signals on the initiation and propagation of childhood absence seizures by power spectrum analysis and network connectivity analysis. This is helpful to investigate dynamic spatial and temporal changes of EEG signals in CAE epileptic seizures. We found that generalized seizures throughout the brain happen within seconds of the onset of seizures in all patients, and the pattern of spatial and temporal evolution is consistent in the range of network connectivity and the distribution of EEG power spectrum. We also compared the EEG power spectrum and synchronization in different frequency bands among the inter-ictal, pre-ictal and ictal states. In this present study, we found excessively elevated PSD and PLV in all frequency bands especially the delta band in the ictal group. Moreover, there are some differences in EEG power and synchronicity between the pre-ictal and inter-ictal groups, suggesting some early warning signals for seizure detection.

### Temporal and spatial dynamic evolution

Electroencephalography power spectrum increased significantly after the onset, reaching a peak of about 2–5 seconds, then decreased due to the attenuation of power (Figure 3). For each patient, EEG power at the frequency band of 2–4 Hz exhibited a clear prevalence during the seizures. This delta band, especially 3 Hz, agrees with the results reported in CAE seizures by the review (Matricardi et al., 2014). In the previous studies, each participant showed the spectral power explosion about the ictal-onset 3-s in CAE (Sun et al., 2020) and the critical source locations from pre-ictal-2-s to onset-2s had been found in CAE generation (Wu et al., 2017). For all 67 seizures in this work, the seizure discharges spread from the local to the global brain area is about 2–5 s, indicating rapid discharges’ diffusion. We study the evolution of EEG power distribution during the seizure process from pre-ictal to postictal states by using the topography shown in Figure 4. The results show that abnormal discharges originate from the local area and have a gradual spatial diffusion process with a specific spatial-temporal evolution pattern, the ranges of spatial diffusion first increases and then decreases. An early topographic EEG research explored the evolution of spike and wave discharges demonstrated that the origin of abnormal discharges was localized (Lemieux and Blume, 1986). Similar spatial diffusion of the discharges has also been observed in previous scalp EEG, EEG-fMRI, and MEG studies (Westmijse et al., 2009; Gupta et al., 2011; Qiu et al., 2017; Leitgeb et al., 2020; Sun et al., 2020). We found that most seizures generate from the frontal-related regions (including frontal, prefrontal, lateral frontal, right frontal, left prefrontal lobes, etc.) as shown in Table 1. The epileptic seizures of 22 patients originated from the frontal area, reaching 66.7% (22/33). The frontal discharges have been observed in these subjects. Furthermore, Figure 4 shows the observed PSD enhancement in the frontal region at the onset of seizures. Studies in patients with absence epilepsy have already reported the importance of the frontal cortex (Szaflarski et al., 2010; Gupta et al., 2011; Sysoeva et al., 2016), and local discharges are mainly predominant in the frontal area (Blume and Lemieux, 1988; Wu et al., 2017). The onset of CAE was characterized by high associations at the left and right frontal regions (Westmijse et al., 2009). Therefore, frontal dominance may play an important role in the initiation of seizures. In addition, we found that the temporal-spatial distribution of PSD was consistent in the continuous multiple episodes of most patients, but varied a lot among different patients. The reason may be that the abnormal discharges in each patient have the same location of origin and transmission route.

Epilepsy is caused by abnormally increased synchronization of brain networks (Fisher et al., 2005). The synchronization of neurons in brain regions may regulate the transmission of information between cortical regions on the coupled temporal and spatial scale, which is essential for consciousness and cognition (Hughes, 2009) and is associated with the transient loss of consciousness in CAE patients. In this article, dynamic network connectivity was performed to explore the interaction among brain regions. Figure 5 shows that the brain network connectivity increases gradually at the onset of a seizure, reaches the maximum, and then declines until it returns to normal, which is consistent with the spatial distribution evolution of EEG power spectral. Particularly, the maxima of these two characteristics almost emerge simultaneously for the same seizure. The essential reason is that the CAE seizures are generated from the local epileptic discharges, and the diffusion leads to a larger range of these abnormal discharges, and then mutual interaction among brain regions results in the enhancement of synchronization. Therefore, the synchronization connectivity of the brain network reaches the strongest when the epileptic discharges spread to the maximum range of the brain area. This result confirms that the spread of CAE is caused by the spread of epileptic discharges and their subsequent interaction with brain regions. Our study confirmed the enhanced synchronization of the brain network and found an intrinsic link between EEG power and brain region synchronization.

### Comparison among inter-ictal, pre-ictal, and ictal groups

The results showed a significant increase of EEG PSD in the ictal group and its distribution in brain area is quite different from the other two groups as shown in Figure 6. The homeostasis system in the brain regulates the excitability of the neurons, keeping the brain activity in the normal range and preventing it from excessive discharges (Stefan and Da Silva, 2013). Any slight disturbance that exceeds the normal range can result in a huge transition to the pathological states, such as seizure or coma. Thus, the results indicated that brain activity is completely altered during the seizure by breaking the homeostasis of the brain. Even for the same type of epilepsy, EEG PSD’s spatial distribution varies greatly among individuals. But for the same patient, similarity occurs in the spatial distribution of multiple consecutive seizures. This could explain the better performances of epilepsy prediction or detection can be achieved by using subject-specific method. Furthermore, Figure 8 that the outliers in the pre-ictal group were overlapped with the main range of the ictal group, revealing that the network connectivity would strengthen at certain moments before the onset. These findings can distinguish between pre-ictal and inter-ictal states, providing a basis for using PSD and its spatial distribution as features for seizure prediction.

The occurrence of the enhanced EEG rhythms seems to be associated with various brain activities or brain diseases. In order to further explore the feature changes of EEG rhythms in CAE patients, this article compared the PSD and PLV of each rhythm among the inter-ictal group, the pre-ictal group, and the ictal group in Figures 7, 9, respectively. Results showed a significant increase of all frequency bands in both PSD and PLV during the seizure period, in line with the results of some previous studies (Piqueira, 2011; Wu et al., 2017; Leitgeb et al., 2020). In this study, we noticed the significantly increased delta and theta power in the pre-ictal group compared to the inter-ictal group. Delta rhythm is the main component during the CAE seizures. The increased delta power in the pre-ictal groups may reflect the generation of epileptic slow-wave discharges before the onset of the seizure. Increased theta activity may reflect frequent discharges before seizures (Li et al., 2018). Glaba et al. (2020) presented the evidence to support the hypothesis that the increased theta rhythm power in CAE patients is a manifestation of cortical hyperexcitability (Vestal and Blumenfeld, 2010). Therefore, we concluded that the coalescence of delta and theta rhythm enhancement is conducive to the occurrence of seizure events in CAE. This interpretation is corroborated by the previous study that found the increased delta and theta power preceded seizures in WAG/Rij rats of absence epilepsy (Sitnikova and van Luijtelaar, 2009). Since some patients in this study used hyperventilation-induced seizures, some previous studies (Clemens, 2004) have shown that hyperventilation can also enhance delta and theta rhythms. Therefore, we performed a statistical analysis of 21 seizures in 10 patients with spontaneous absence seizures (shown in Supplementary material). It was found that the phenomenon of delta and theta rhythm enhancement still existed before the seizure onset.

In terms of PLV, the synchronization of delta rhythm decreased while the alpha rhythm increased in the pre-ictal group compared with the inter-ictal group. Altered rhythm regulations were demonstrated relating to diffuse activation and hypersynchrony in patients with epilepsy (Clemens, 2004). Delta diffusion exists before the epileptic seizure, leading to the enhanced delta rhythm power. However, the synchronization of delta rhythm decreased in the pre-ictal states. Therefore, the results indicated that the frequency of delta rhythm is increased but the occurrence of delta rhythm is not synchronous in each electrode. Alpha rhythm, associated with the remarkable inhibitory effects (Qin et al., 2020; Pellegrino et al., 2021), can reduce cortical excitability (Sauseng et al., 2009) and maintain the functional integrity of brain networks (Waldhauser et al., 2012; Pellegrino et al., 2021), which is a safety mechanism to protect the brain and prevent transition to seizure (De Curtis and Avanzini, 2001; Badawy et al., 2010). Our results showed increased synchronization of alpha rhythm in the pre-ictal group, while there was no difference in alpha PSD between the inter-ictal and pre-ictal groups. Therefore, synchronization of alpha rhythm is a good feature to classify these two groups.

### Limitations

Several limitations should be considered in the present study. Firstly, EEG with low spatial resolution is hard to detect the exact profile of the spatial location. Thus, in the future, we will combine with other multi-modal imaging technologies to improve spatial resolution. Secondly, CAE is a kind of age-dependent epilepsy. Children’s age has a great influence on EEG signals in CAE patients, but the relatively small sample size in the current study is not amenable to further subdivision according to different age groups. Lastly, the results may be affected by artifacts from EEG signals, although a filter has been used to minimize these artifacts. Further work, these factors will be considered to obtain more reliable results.

## Conclusion

Combining time-frequency and time-space analysis of the EEG provides comprehensive information to describe the dynamic spatial and temporal changes in CAE patients. Despite the fact that CAE is classified as a generalized type of epilepsy, we confirmed the existence of a rapid diffusion process (within seconds) in the localized pattern of activation with frontal dominance. Our results found that the patterns in the distribution of EEG PSD are consistent with that in the spatial synchronization connectivity during the seizures, suggesting that the initiation and propagation of CAE seizures are the results of the combination of the abnormal discharges diffusion and brain regions synchronization. In terms of the comparison among inter-ictal, pre-ictal, and ictal groups, the ictal group is different from the other two groups, herein brain activity is completely changed. Our study also confirmed that epileptiform discharges may occur prior to epileptic seizures due to the increased delta and theta power in the pre-ictal group, which seem to be good biomarkers. Individual differences bring difficulties to the clinical diagnosis and detection of epilepsy. Still, the similarity of the EEG PSD spatial distribution among multiple seizures in the same sample can be used to predict epilepsy clinically. Taken together, our results provide a new window on the dynamic spatial and temporal evolution of EEG signals in CAE and contribute to understanding the origin and transmission mechanism.

## Data availability statement

The raw data supporting the conclusions of this article will be made available by the authors.

## Ethics statement

The studies involving human participants were reviewed and approved by the Ethical Committee of the Children’s Hospital of Chongqing Medical University. Written informed consent to participate in this study was provided by the participants or their legal guardian/next of kin.

## Author contributions

LSZ, JW, and ZYL designed the work and wrote this original manuscript. SLH, JZW, and XFZ contributed to the acquisition and analysis of data. LSZ and ZWH contributed to the review and editing. ZYL was mainly responsible for this project. All authors contributed to the article and approved the submitted version.
