# Supplementary material for: Temporal and spatial dynamic propagation of electroencephalogram by combining power spectral and synchronization in childhood absence epilepsy
Source: Front Neuroinform. 2022 Aug 16;16:962466. doi: 10.3389/fninf.2022.962466 (PMC9433125; doi:10.3389/fninf.2022.962466)
Supplement: Supplementary file 1 [file Data_Sheet_1.docx]

Figure 1 single-subject’s PSD topographies under three states.

Figure 2 a statistical analysis of 21 seizures in 10 patients with spontaneous absence seizures (P<0.05).
